# Probenecid treatment improves outcomes in a novel mouse model of peripartum cardiomyopathy

**DOI:** 10.1371/journal.pone.0230386

**Published:** 2020-03-27

**Authors:** Evan Onusko, Michael R. McDermott, Nathan Robbins, Guansheng Liu, Evangelia G. Kranias, Jack Rubinstein, Sheryl E. Koch

**Affiliations:** 1 Division of Cardiovascular Health & Disease, Department of Internal Medicine, University of Cincinnati College of Medicine, Cincinnati, Ohio, United States of America; 2 Department of Pharmacology & Systems Physiology, University of Cincinnati College of Medicine, Cincinnati, Ohio, United States of America; Scuola Superiore Sant'Anna, ITALY

## Abstract

Probenecid has been used for decades in the treatment of gout but recently has also been found to improve outcomes in patients with heart failure via stimulation of the transient receptor potential vanilloid 2 (TRPV2) channel in cardiomyocytes. This study tested the use of probenecid on a novel mouse model of peripartum cardiomyopathy (PPCM) as a potential treatment option. A human mutation of the human heat shock protein 20 (Hsp20-S10F) in mice has been recently shown to result in cardiomyopathy, when exposed to pregnancies. Treatment with either probenecid or control sucrose water was initiated after the first pregnancy in both wild type and Hsp20-S10F mice. Serial echocardiography was performed during subsequent pregnancies and hearts were collected after the third pregnancies for staining and molecular analysis. Hsp20-S10F mice treated with probenecid had decreased mortality, hypertrophy, TRPV2 expression and molecular parameters of heart failure. Probenecid treatment also decreased apoptosis as evidenced by an increase in the level of Bcl-2/Bax. Probenecid improved survival in a novel mouse model of PPCM and may be an appropriate therapy for humans with PPCM as it has a proven safety and tolerability in patients with heart failure.

## Introduction

Peripartum cardiomyopathy (PPCM) is a potentially life-threatening disease in which the cardiac function of the mother drops significantly between the last month of pregnancy and the first months postpartum [1]. Though as there is no specific test to confirm the disease, it remains a diagnosis of exclusion in women presenting with an idiopathic cardiomyopathy “towards the end of pregnancy or in the months following delivery, abortion or miscarriage, without other causes for heart failure, and with a left ventricular (LV) ejection fraction (EF) < 45%” as per the latest position statement from the European Society of Cardiology [2]. It often presents with development of dilated cardiomyopathy (DCM) and can present with variable degrees of signs and symptoms of decreased cardiac function and increased natriuretic peptides consistent with heart failure with reduced ejection fraction (HFrEF), though in contrast to DCM the prognosis for PPCM is likely better as long as subsequent pregnancies are avoided [1,3–5]. The disease has significant implications for the patient’s quality of life as subsequent pregnancies are strongly linked to deteriorating cardiac function and are contraindicated [6]. PPCM is diagnosed in 1 in 2000 to 4000 live births in the United States [7,8] and more frequently in other parts of the world such as Nigeria and Haiti, likely due to cultural and genetic risk factors [9,10]. Both of these findings possibly underestimate the actual incidence of PPCM, as some of its findings may be confused with normal physiologic changes of pregnancy [10]. Even with current medical therapy, the outcome for patients with PPCM is still suboptimal [11].

Like other forms of HFrEF, PPCM is usually treated with renin-angiotensin inhibitors, beta-blockers, diuretics, and nitrates [10], as there is no disease-specific therapy available for PPCM, with the possible exception of bromocriptine [12]. The relatively few advancements in treatment of PPCM can be partially explained by the paucity of appropriate animal models to study this disease [13].

While studying a naturally-occurring polymorphism in the Hsp20 gene (S10F), we recently discovered that this mutation not only diminished the cardioprotective effects of Hsp20 in a transgenic mouse model, but mutant female mice also developed DCM during the course of 2 to 4 pregnancies that resulted in the death of 70% after three pregnancies and 100% after the fourth [14]. It is well known that Hsp20 is a small heat shock protein that exhibits cardioprotective effects via inhibition of several signaling cascades that result in hypertrophy, apoptosis, and myocardial ischemia [15–18]. Levels of total and phosphorylated Hsp20 are known to increase in patients with dilated and ischemic cardiomyopathy [19] and mutations of the protein have been found in DCM patients in various populations [20–22].

This Hsp20-S10F mouse model of PPCM demonstrated increasingly severe signs of DCM after multiple pregnancies, consistent with the classic presentation of PPCM. In addition to the decreased survival, there was an increase in LV end systolic and diastolic volume and a decreased EF. Molecular effects included an increase in atrial naturietic peptide (ANP), beta natriuretic peptide (BNP), and Caspase-3 activity. Thus, it was proposed that the mouse containing the Hsp20 S10F mutation would make a valid and potentially clinically relevant model of PPCM to study various therapeutic options [14].

As the Hsp20 S10F mutation is also associated with impaired calcium handling and increased apoptosis by impeding Hsp20’s interaction with the proapoptotic protein Bax [14], we hypothesized that transient receptor potential vanilloid 2 (TRPV2) stimulation may play a protective role in the development of PPCM in this mouse model. The TRPV2 channel is a stretch and agonist-activated calcium channel [23]. Our laboratory has previously described that stimulation of TRPV2 with an agonist, probenecid, results in positive inotropic effects through an increase in cytosolic calcium via calcium-induced calcium release. Furthermore, other studies have also shown that probenecid can increase apoptosis via inhibition of pannexin-1 channels (PANX1) but not through Bax [24,25]. These studies have been conducted in Jurkat cells and in ex vivo Langendorff rat hearts. Conversely, our own mouse studies have demonstrated that probenecid does not increase the rate of apoptosis even in the context of improved inotropism [26,27]. Therefore, the purpose of this study was to determine if the TRPV2 agonist probenecid could have an effect on this novel PPCM mouse model [14].

There are important clinical implications of our study, as probenecid has been safely used in children and adults as an adjunct to treatment with antibiotics and for the prevention of gout for decades [28] and has been used throughout the first trimester with no reported congenital birth defects. It has also been shown to have no adverse effects on fetal development, making it an attractive and easily translatable treatment for PPCM [29].

In order to test the hypothesis, we treated wild-type and Hsp20-S10F mutant mice with oral probenecid and measured the cardiac function using echocardiography at various time points before, during, and after pregnancies and compared the survival of control and treated mice over the course of three pregnancies.

This study demonstrates that probenecid therapy improved survival in a novel mouse model of peripartum cardiomyopathy, likely through modulation of calcium handling, though the exact mechanism of action in this model will be the subject of further study.

## Materials and methods

### Animals

**Fig 1:**
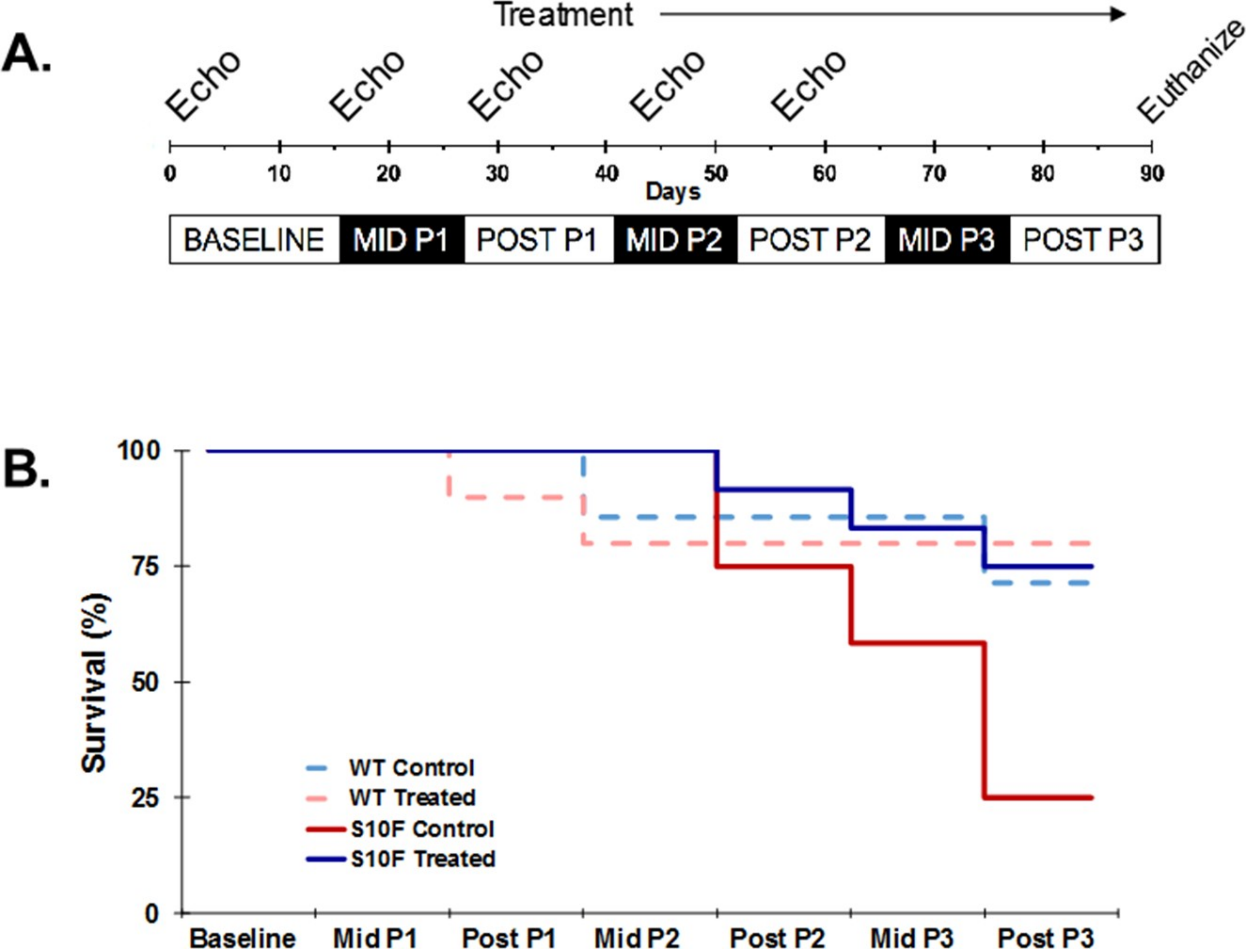
Hsp20-S10F study timeline and survival curve. **(A)** Schematic timeline of the three pregnancies for WT and Hsp20-S10F mice. **(B)** Kaplan Meier mouse survival curve for WT control (n = 14/4), WT treated with probenecid (n = 10/2), Hsp20-S10F control (n = 12/9), and Hsp20-S10F treated with probenecid (n = 12/3).

All animal procedures were performed with the ethical and technical approval of the Institutional Animal Care and Use Committee (IACUC) of the University of Cincinnati and in accordance with the Eighth Edition of the Guide for the Care and Use of Laboratory Animals [30]. All lab members involved in animal research received specialized training from Laboratory Animals Medical Services, the centralized animal care program at the University of Cincinnati. Animal data included in this study was obtained over the years 2015 to 2019. In addition, the following criteria were used as indications for immediate euthanasia: respiratory distress/failure, aberrant inactivity with obvious discomfort, severe apparent pain and/or recommendations from vivarium veterinarian staff. If any of these criteria were met, the animal was immediately euthanized in the interest of minimizing suffering. Euthanasia was performed using medical- or technical-grade compressed CO_2_. The displacement rate was 10–30% of the chamber cage volume/minute regulated by a flow meter. Consistent with the above guidelines, housing and nesting material were included in cages, and any apparent discomfort was immediately reported with appropriate intervention; analgesics or anesthetics were not utilized during this study. Animals were observed by a lab member at least once every 24 hours and by an animal technician at least once every 12 hours. In total, 3 animals were euthanized due to severe respiratory distress secondary to heart failure; 15 animals were found dead due to apparent heart failure; 30 were euthanized for analysis at proposed endpoint of study. The generation and initial characterization of the Hsp20-S10F mice (FVB/n background) were previously described [14]. WT and Hsp20-S10F FVB/n littermate female mice aged 10–12 weeks had echocardiography measurements taken as baseline parameters. After echocardiography, age matched WT males (FVB/n) were added to individual cages with either an Hsp20-S10F or WT female. All mice were kept in the same room, with a 12/12 hour light/dark cycle with unlimited access to food and water. Mid pregnancy (Mid P1 and Mid P2) measurements were taken between day embryonic day 14 (E14) and 18 (E18). Post pregnancy (Post P1 and Post P2) measurements were taken 7–10 days after delivery (See Fig 1A).

### Echocardiography

All echocardiographic studies were performed as previously described [26]. Briefly, mice were anesthetized with isoflurane and placed on the heated stage of the Vevo 2100. Parasternal long axis (PSLAX) and short axis (SAX) images were recorded and then analyzed on a separate work station with VevoStrain software (Vevo 2100, v1.6, Visualsonic, Toronto, Canada). From the M-mode images, left ventricular cavity size for systole and diastole (LV dia;s, LV dia;d, LV Vol;s and LV Vol;d) were measured. Heart rate (HR), stroke volume (SV), and the echocardiographic calculation for fractional shortening (FS), ejection fraction (EF) and cardiac output (CO) were obtained using the Vevo software. Treatment and control groups were assessed on the same day by the same technician in order to minimize variation.

### Speckle tracking measurements

Speckle tracking based displacement and strain values were calculated from the SAX view for each individual wall segment using VevoStrain software (Vevo 2100, Version 1.1.1 B1455, VisualSonics) as previously described [31]. Briefly, a single blinded reader loaded the B-mode cine loop into VevoStrain software and ECG measurements taken synchronously with the B-mode cine loop were used to select three to four cardiac cycles out of the cine loop. An M-mode trace across the heart of the selected cardiac cycles was visually inspected to qualitatively confirm that ultrasound imaging of heart contractility matched the ECG data. A frame during diastole was selected, and the endocardial border was traced by the observer. The epicardial border was automatically selected by the software and subsequently confirmed by the observer. The VVI software was initiated to compute the radial displacement. Appropriate speckle tracking was verified by manual inspection of the software-determined movement of the traced borders over three cardiac cycles. B-mode cine loops were selected to minimize image artifacts and ensure good visualization of the borders.

### Probenecid treatment

After the Post P1 measurement, both WT and Hsp20-S10F mice were randomized to probenecid-treated sucrose water or untreated sucrose water as control. To avoid subjective bias, mice were randomized via numbers assigned at birth; team members were blinded to Post P1 measurements until treatment groups had been assigned. Appropriate sample size was determined for multiple independent study groups with dichotomous endpoints (survival vs mortality). By extrapolating expected mortality, a sample size of 12 was determined to provide a power of 85% when alpha value was set to 0.05. 48 mice were used in total with 12 assigned to Hsp20-S10F treatment group, 12 to Hsp20-S10F control, 14 assigned to WT control, and 10 to WT treatment; each mouse was treated as a single experimental unit for analysis. Despite our efforts to maintain similar sample sizes, the mortality associated with the PPCM model caused the actual sample sizes to be different at the last time point. Probenecid was dissolved in water containing 5% sucrose at a concentration of 0.5 mg/mL. Both probenecid-treated and sucrose water were administered via bottles in the animals’ home cage to minimize intrusion and ensure that all treatment groups were receiving treatment simultaneously. Water was changed twice a week, and the volume was measured before and after the consumption to determine the approximate dose. The calculated daily probenecid dose was 108.64±6.26 mg/kg/day. The primary experimental outcome for this project is mortality rate with secondary outcomes including cardiac function (including displacement/strain), ANP and TRPV2 mRNA, and TRPV2, Bcl-2/Bax, and p-Akt/t-Akt protein expression.

### Real-time quantitative RT-PCR (qRT-PCR)

Total RNA was isolated (Qiazol method, Qiagen, Venlo, Limburg, Netherlands) and cDNA synthesized (High Capacity RNA-to-cDNA kit, Applied Biosystems, Carlsbad, CA) per manufacturer’s instructions. Expression levels were determined using the following primer pairs: TRPV2, sense 5’- CTACTGCTCAACATGCTC-3’ and anti-sense 5’- CTCATCCAGGTATACCATCC-3’, atrial natriuretic peptide (ANP), sense 5’- GGGGGTAGGATTGACAGGAT and anti-sense 5’- CAGAGTGGGAGAGGCAAGAC, and vascular endothelial growth factor A (VEGF), sense 5’-TTACTGCTGTACCTCCACC and antisense, 5’-ACAGGACGGCTTGAAGATG. The primer pairs for GAPDH were sense, 5’-CATGGCCTTCCGTGTTCCTA-3’ and anti-sense 5’-CCTGCTTCACCACCTTCTTGAT-3’. All PCR experiments were performed as described[26].

### Western blot analysis

Protein was isolated from heart samples (n = 4 for each group), as previously described[32]. Briefly, heart samples were homogenized in the following buffer: 20mM HEPES (pH 8.0), 150mM sodium chloride, 1% sodium deoxycholate, 1% sodium dodecyl sulfate, protease inhibitor cocktail (P8340; Sigma Aldrich, St. Louis, MO) and phosphatase inhibitor cocktail (EMD, Merck, Darmstadt, Germany) and centrifuged at 100,000xg for 20 minutes. Protein concentration and western blot analysis were conducted as previously described [26]. For each sample, 100μg of protein was loaded on the same 10% gel (Criterion gel, Bio-Rad, Hercules, CA) for TRPV2 (AB5398, Millipore, Bellerica, MA). Similarly, 50μg of protein of each sample was loaded on 12% gels (Criterion gel, Bio-Rad, Hercules, CA) for Bax (Cell Signaling Technologies, 2772), Bcl-2 (Santa Cruz Biotechnolgies, sc-783), p-Akt (Cell Signaling Technologies, 9271) and total-Akt (t-Akt, Cell Signaling Technologies, 9272). The blots were stripped (Restore Western Blot Stripping Buffer, ThermoScientific, Rockford, IL) and normalized with GAPDH (sc-25778, Santa Cruz Biotechnologies, Santa Cruz, CA). Proteins bands were visualized using Western Lightning reagents (PerkinElmer, KY) and the FluorChemE (ProteinSimple, CA). The densitometry of the bands was determined using AlphaEase software (ProteinSimple formally Alpha Innotech, CA) and normalized to GAPDH for loading control.

### Connective tissue analysis

Hearts were fixed in 4% paraformaldehyde overnight and paraffin embedded by the CCHMC Department of Pathology Research Core (Cincinnati, Ohio). The paraffin-embedded blocks were cut into 6μm sections and mounted on slides. Longitudinal heart sections were stained with Masson’s Trichrome and images from the left ventricle were analyzed using the Image J software as previously described [31]. The average fibrotic area for each group was determined and each group was analyzed using student t-test.

### Statistical analysis

Statistical analysis was performed with SigmaStat, now incorporated into SigmaPlot v.13.0. Unit of analysis for each dataset was treatment vs control treatment group with further distinction between WT or Hsp20-S10F. Data were tested for normality and equal variance. For two-group comparison of parametric data a Student’s t-test was performed, while statistical significance between multiple groups was assessed by analysis of variance (ANOVA) for one-way or two-way mixed design with repeated measures as appropriate. Where significance was indicated, post-hoc testing was performed using the Holm-Sidak method for comparing individual means and correcting for family-wise error or the Fisher-LSD method for all pairwise multiple comparison procedures (SigmaPlot v.13.0, Systat Software, Inc., San Jose, CA). Data are presented as means ± S.E.M., and differences were regarded as significant at P ≤ 0.05. Percent survival is plotted on the Kaplan-Meier graph and significance of the trends was determined using Log-rank (Mantel-Cox) Test.

## Results

### Baseline data revealed no significant differences between groups

All experimental groups showed no significant difference among primary or secondary experimental outcomes, including cardiac function, survival prior to treatment, or exposure to prior testing (Table 1).

**Table 1:** Mouse echocardiographic data from WT control, WT treated with probenecid, Hsp20 S10F control, and Hsp20 S10F treated with probenecid at baseline.

Baseline	WT Control	WT Treated	S10F Control	S10F Treated
	n = 13	n = 8	n = 8	n = 14
**Heart Rate**	397.38±15.58	398.38±13.94	384.00±27.74	393.00±14.42
**Systolic Diameter**	2.56±0.06	2.75±0.08	2.89±0.15	2.87±0.09
**Diastolic Diameter**	3.87±0.06	3.93±0.08	4.09±0.11	3.96±0.06
**LV Systolic Volume**	23.94±1.42	28.64±2.1	33.14±4.21	31.95±2.32
**Stroke Volume**	41.06±1.14	38.90±2.01	41.50±1.49	36.86±0.9
**Fractional Shortening**	33.99±0.77	30.05±1.18	29.67±1.9	27.81±1.13
**Cardiac Output**	16.38±0.89	15.7±1.33	15.70±0.84	14.47±0.68

WT, wild type mice; S10F, Hsp20 S10F mice; LV, left ventricle.

### Probenecid improved survival in Hsp20-S10F mice

Survival of control and treated WT and Hsp20-S10F female mice was compared during the course of three pregnancies. As shown in Fig 1B, treatment with probenecid greatly increased the likelihood of survival of Hsp20-S10F mice (75%; n = 9/12) when compared to control Hsp20-S10F mice (25%; n = 3/12). All Hsp20-S10F mice survived the first pregnancy, though 3/12 control Hsp20-S10F mice died after the second pregnancy and 6/9 of the surviving mice died immediately following or during the third pregnancy. The likelihood of survival of the treated Hsp20-S10F mice, 75%, was comparable to those of control (10/14) and treated (8/10) WT mice (71% and 80% survivability, respectively P = 0.05). We observed that the WT control mice had a higher than expected mortality rate, which may have been secondary to the stress induced by frequent adding and removing of males.

### Cardiac function was preserved in treated Hsp20-S10F mice

**Fig 2:**
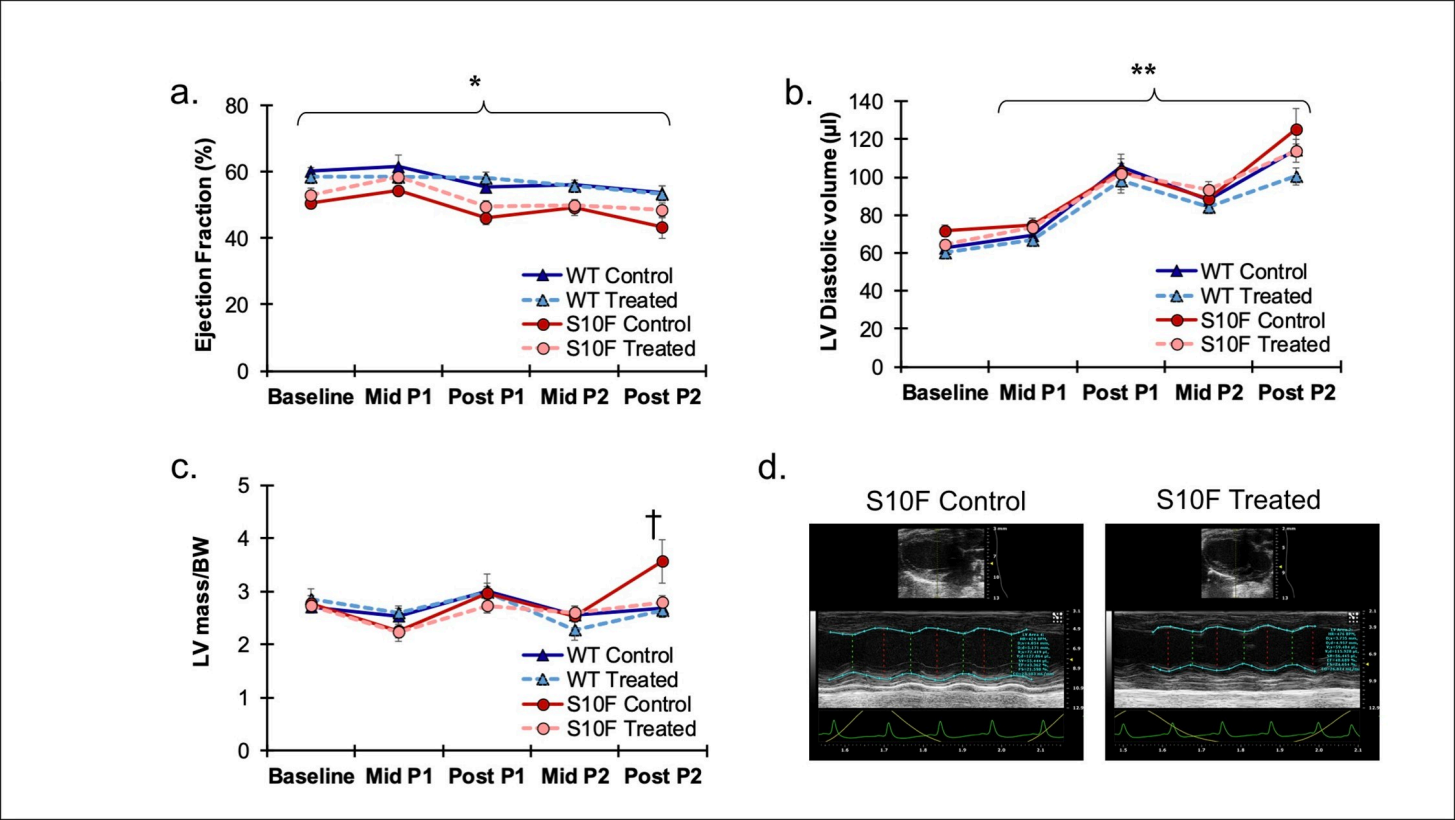
Measurement of echocardiography parameters in Hsp20-S10F mice. Echocardiographic measurement of ejection fraction (EF). **(A)** Longitudinal echocardiographic study of the EF for WT control (n = 13), WT treated with probenecid (n = 8), Hsp20-S10F control (n = 8), and Hsp20-S10F treated with probenecid (n = 14). *P = 0.015 for WT mice (control and treated) compared to Hsp20-S10F mice (control and treated). Two-way ANOVA repeat measures. **(B)** Longitudinal echocardiographic study of the LV diastolic volume for WT control, WT treated with probenecid, Hsp20-S10F control, and Hsp20-S10F treated with probenecid. **P<0.001 for all four groups compared to baseline. Two-way ANOVA repeat measures. **(C)** Longitudinal echocardiographic study of LV mass normalized to body weight for WT control, WT treated with probenecid, Hsp20-S10F control, and Hsp20-S10F treated with probenecid. †P = 0.049 for Hsp20-S10F control versus WT control, WT treated and Hsp20-S10F treated mice. Two-way ANOVA repeat measures. **(D)** Representative B-mode and M-mode echocardiographic images.

Echocardiographic assessment demonstrated a decrease in systolic function in all groups over time as measured via EF. Further, the EF was higher in WT control (n = 13) and WT treated (n = 8) mice compared to Hsp20-S10F control (n = 8) and Hsp20-S10F treated (n = 14) mice (P = 0.015). There was no difference in EF between the WT control versus WT treated or Hsp20-S10F control versus Hsp20-S10F treated groups (Fig 2A). As shown in Fig 2B, all four groups investigated had an increase in LV diastolic volume as measured at each time point compared to baseline (P<0.001). Treatment with probenecid did not have an effect on the increase in LV diastolic volume for any group of mice Post P2. LV mass, normalized to body weight (LV mass/BW) remained unchanged between all the groups until Post P2, where the Hsp20-S10F untreated mice had a significant increase compared to the other 3 groups (P = 0.049, Fig 2C, with representative images in Fig 2D).

### Speckle tracking measurements demonstrated minimal changes in cardiac function

**Fig 3:**
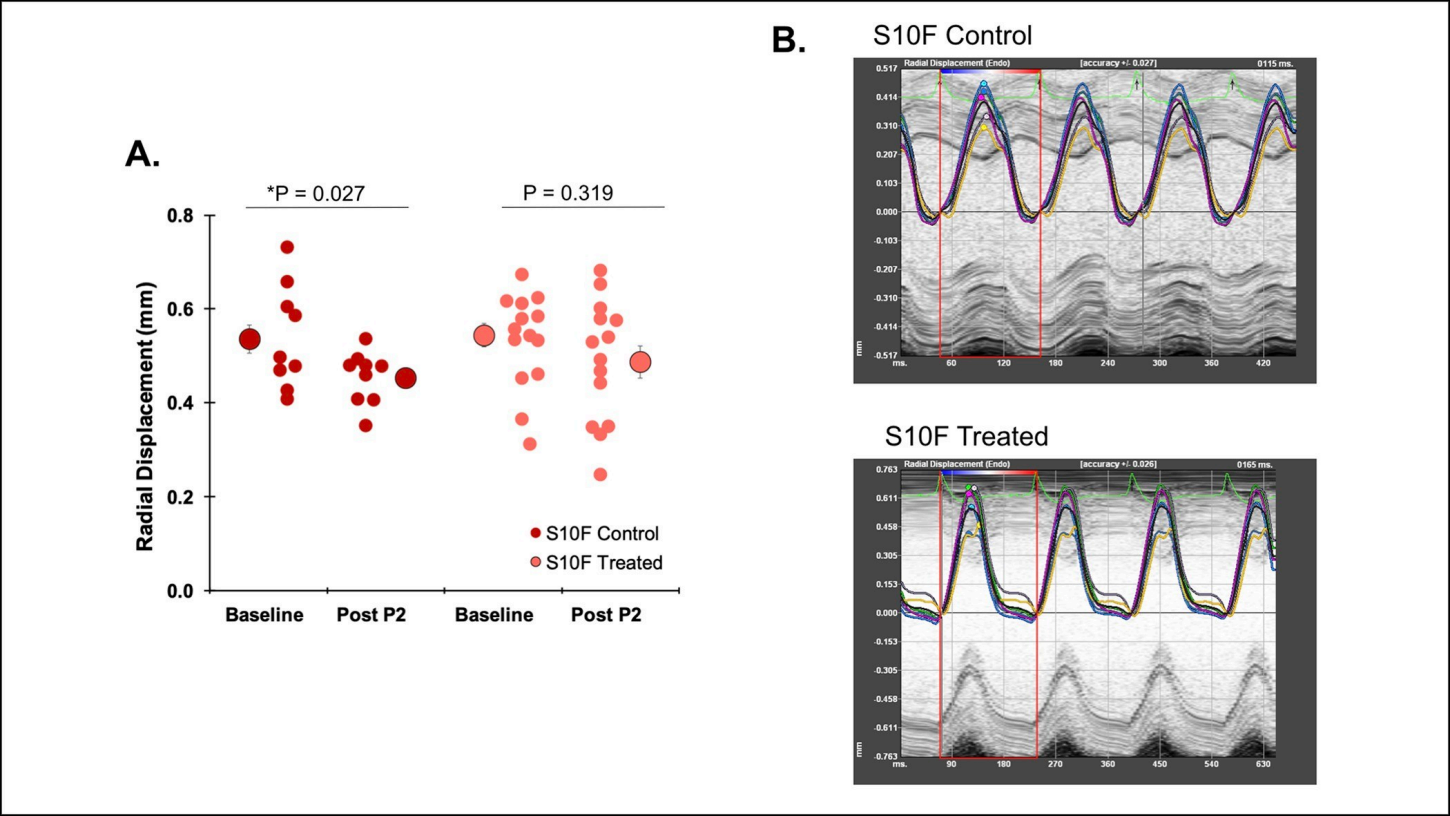
Measurement of advanced echocardiography parameters in Hsp20-S10F mice. Radial displacement determined from speckle tracking derived data. **(A)** Radial displacement (mm) measured at baseline and Post P2 for Hsp20-S10F control and treated mice. *P = 0.027 for Hsp20-S10F control mice. Paired Student’s t-test. **(B)** Representative radial displacement images for Hsp20-S10F control and treated mice.

Speckle tracking derived data demonstrated a trend toward a decrease in radial displacement in the Hsp20-S10F control group from baseline to Post P2 (0.54±0.03 and 0.45±0.02; P = 0.027, n = 9) that was not observed in the treated group (0.54±0.03 and 0.49±0.03; P = 0.319, n = 14) (Fig 3A with representative images in Fig 3B). Probenecid did not have a significant effect on the radial displacement of the WT control (0.53±0.19 and 0.57±0.038, n = 11) and WT treated (0.52±0.86 and 0.58±0.39, n = 7) mice from baseline to Post P2, respectively.

### Probenecid treatment blunted the cardiac dysfunction of treated Hsp20-S10F mice in comparison to the dysfunction found in the Hsp20-S10F control mice

There was no significant difference between the groups at baseline. Almost every echocardiographic parameter measured changed from baseline (Table 1) to Post P2 (Table 2). Treatment with probenecid mitigated some of the effects of pregnancy in both the control and Hsp20-S10F mice, though not significantly. The Hsp20-S10F treated mice, while still having a decrease in cardiac function compared to the WT control and WT treated mice, had improved function as compared to the Hsp20-S10F control mice, as evidenced by a decreased heart rate, and decreased systolic volume. Table 2 lists the full comparison of the echocardiographic data from baseline to Post 2. Statistical comparisons were made between the WT control and Hsp20-S10F control mice, as well as between WT treated and Hsp20-S10F treated mice. The only significant change, with the exception of EF, diastolic volume and LV mass/BW (as described above and in Fig 2) was the decrease in FS in the Hsp20-S10F control as compared to WT control (P = 0.043).

**Table 2:** Mouse echocardiographic data from WT control, WT treated with probenecid, Hsp20 S10F control, and Hsp20 S10F treated with probenecid at Post P2.

Post-Pregnancy 2	WT Control	WT Treated	S10F Control	S10F Treated
	n = 13	n = 8	n = 8	n = 14
**Heart Rate**	444.77±9.50*	446.13±20.29	427.22±13.85	397.29±17.65
**Systolic Diameter**	3.43±0.15*	3.24±0.14*	3.88±0.14*	3.62±0.16*
**Diastolic Diameter**	4.82±0.12*	4.63±0.16*	5.08±0.15*	4.93±0.13*
**LV Systolic Volume**	50.12±5.29*	43.02±4.68	66.36±5.75*	57.15±6.35*
**Stroke Volume**	59.85±2.21*	56.62±4.71*	57.88±3.69*	58.65±4.00*
**Fractional Shortening**	29.29±1.47*	30.00±1.74	23.69±1.14*†	26.79±1.79
**Cardiac Output**	26.63±1.15*	25.38±2.48*	24.481.33*	23.02±1.53*

WT, wild type mice; S10F, Hsp20 S10F mice; LV, left ventricle

*P<0.05 compared to baseline (Table 1).

†P<0.05 compared to WT control Post P2.

Two-way ANOVA, post-hoc Fisher LSD method for all pairwise multiple comparisons.

### mRNA expression of ANP was increased in the Hsp20-S10F control mouse and blunted with probenecid treatment

**Fig 4:**
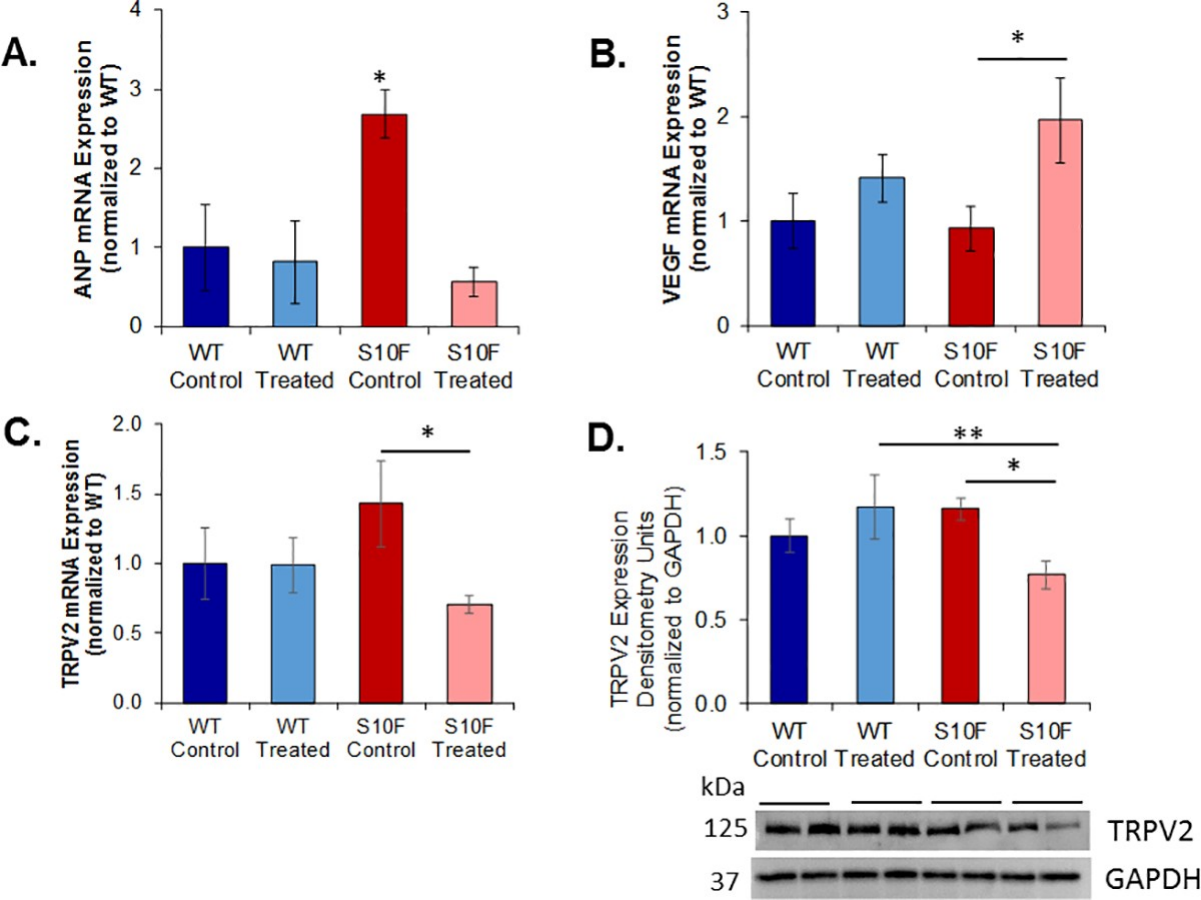
Molecular analysis of mRNA and protein. qRT-PCR and western blot analysis of WT control, WT treated with probenecid, Hsp20-S10F control, and Hsp20-S10F treated with probenecid (n = 4/group) mice. **(A)** ANP mRNA expression normalized to WT control. *P<0.002 for Hsp20-S10F control compared to all other groups. **(B)** VEGF mRNA expression normalized to WT control. *P = 0.026 for Hsp20-S10F treated compared to Hsp20-S10F control. (**C**) TRPV2 mRNA expression normalized to WT control. *P = 0.035 for Hsp20-S10F treated compared to Hsp20-S10F control. **(D)** Protein expression of TRPV2 normalized to GAPDH. *P = 0.039 for Hsp20-S10F treated compared to Hsp20 S10F control, **P = 0.035 for Hsp20-S10F treated compared to WT treated. Two-way ANOVA, post-hoc Fisher LSD method for all pairwise multiple comparison procedures.

The Hsp20-S10F control mice demonstrated a marked increase in mRNA expression of ANP compared to WT control (2.7 fold) and Hsp20-S10F treated (4.8 fold) mice. Importantly, probenecid treatment of the Hsp20-S10F control mice resulted in decreased ANP expression levels, which were comparable to WTs (Fig 4A).

### mRNA expression of VEGF was increased in the probenecid treated HSP20-S10F mouse

The VEGF mRNA expression was not different between the WT control, WT treated and S10F control mice. The Hsp20-S10F treated mice had an increase in expression compared to the Hsp20-S10F control (2.1 fold) (Fig 4B).

### mRNA and protein expression of the TRPV2 channel was decreased in the Hsp20-S10F probenecid treated mouse

The stretch activated TRPV2 channel demonstrated a slight, but not significant, increase in mRNA expression in the Hsp20-S10F control compared to WT control and WT treated mice. Treatment with probenecid in the HSP20-S10F mice significantly decreased TRPV2 mRNA expression relative to the Hsp20-S10F control (P = 0.035; Fig 4C). Similarly, the protein expression of TRPV2 was decreased in the Hsp20-S10F treated mice compared to Hsp20-S10F control mice (P = 0.039) and WT treated mice (P = 0.039, Fig 4D).

### Differences in markers of apoptosis among the groups

**Fig 5:**
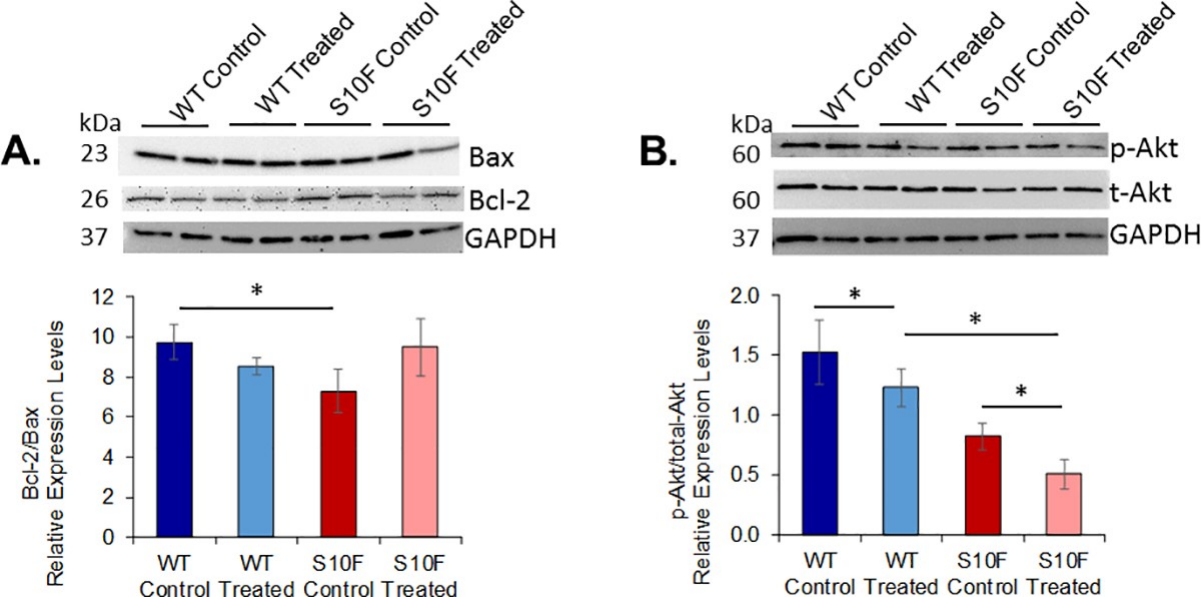
Determination of apoptotic proteins. Western blot analysis of WT control, WT treated with probenecid, Hsp20 S10F control, and Hsp20 S10F treated with probenecid (n = 4/group) mice. **(A)** Quantitative Bcl-2/Bax ratio after normalization to GAPDH. *P = 0.039 for WT control compared to Hsp20-S10F control. **(B)** Quantitative p-Akt/t-Akt ratio after normalization to GAPDH (*P<0.05). Two-way ANOVA, post-hoc Fisher LSD method for all pairwise multiple comparison procedures.

Expression levels of proteins involved in apoptosis were investigated to determine if probenecid treatment blunted these pathways. The Hsp20-S10F control mice had a decreased (P = 0.039) ratio of Bcl-2/Bax as compared to WT control, indicating an increase in this apoptotic pathway (Fig 5A). Levels of p-Akt relative to t-Akt (which also mediates cardiac hypertrophy), were significantly decreased in the WT treated compared to the WT control mice (P = 0.014) and in the Hsp20-S10F treated and compared to the Hsp20-S10F control mice (P = 0.012, Fig 5B).

### Probenecid treatment did not have an effect on connective tissue formation

**Fig 6:**
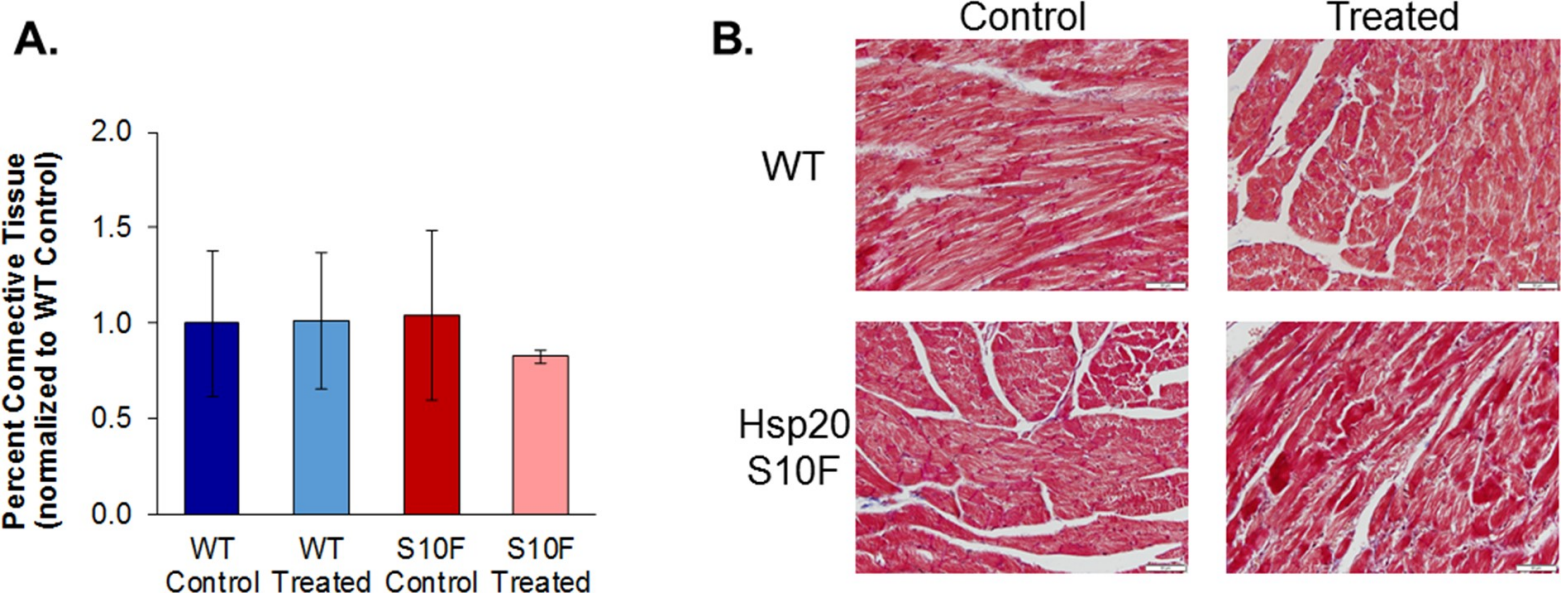
Histological analysis of mouse heart. Fixed heart tissues from WT control, WT treated with probenecid, Hsp20-S10F control, and Hsp20-S10F treated with probenecid (n = 4/group) mice stained with Masson’s Trichrome for **(A)** percentage connective tissue and **(B)** representative images of stained slides.

Masson’s Trichrome stained slides indicated that there was a decrease (that was not statistically significant) in the amount of fibrosis in the Hsp20-S10F treated group as measured by the percent of connective tissue in comparison to the other three groups Post P2 (Fig 6A with representative images in Fig 6B).

## Discussion

In this study, we demonstrate for the first time that probenecid treatment improves outcomes in a mouse model of PPCM, induced via the human Hsp20 S10F mutation. Other mouse models of PPCM have been previously reported and used to test novel therapeutic options for patients with this disease. Most notably, the lack of peroxisome proliferator-activated receptor gamma coactivator 1-alpha (PGC-1α) caused a disruption in both cardiac angiogenesis pathways, vascular endothelial growth factor (VEGF) and prolactin, leading to PPCM. Treatment with VEGF proteins and bromocriptine prevented cardiac dilation and left ventricular dysfunction [8,33]. Furthermore, G alpha(q) cardiac overexpression mice that had increased apoptosis and a PPCM phenotype also had improved outcomes and cardiac function when caspase activity was inhibited [34]. The mouse model used in the current study [14] shares several characteristics with these mouse models including the development of cardiac dilation as well increased apoptosis. Lastly, others have reported that prolactin and angiogenesis associated molecules such as cathepsin D (cleaves prolactin) and microRNA-146a (downstream mediator of a prolactin fragment) also mediate the development of PPCM in a cardiac specific Stat3-/- mouse model [35,36]. The mouse model used in this study also shares several important phenotypes with the human presentation of the disease, including otherwise normal function and survival, when the female mice are not exposed to pregnancy and worsening function and death when exposed to subsequent pregnancies. Further comparison among models is limited, as the underlying pathology of PPCM is not well understood.

Probenecid has been previously demonstrated to improve outcomes in an animal model of ischemic cardiomyopathy [27], in patients with HFrEF [37]. More recently, in a large population study of almost 40,000 elderly patients that were treated for gout, probenecid therapy was found to be associated with decreased risk of heart failure and heart failure related hospitalization (as well as decreased incidence of myocardial infarction and stroke) in comparison to patients treated with allopurinol [38]. Moreover, probenecid has a long clinical record of safety in the general population with minimal side effects [39] and case reports of its use throughout pregnancy have not shown adverse fetal effects [40]. Lastly, the estimated dose administered in this study was approximately 100 mg/kg/day, which when scaled allometrically is lower than the standard humane dosage of 1 gram orally twice per day (for a 70 kg. patient) [41]. This dose is consistent with our prior publication that demonstrated that the 100mg/kg dose was sufficient for increasing EF in mice [26,27].

In this study, cardiac function was assessed serially in both the WT and the Hsp20-S10F mutant female mice throughout the pregnancies. In contrast to a previous study in these mice [14], we attempted to obtain echocardiography during and between pregnancies. This necessitated removing male partners to prevent back-to-back pregnancies. In this respect, our studies took much longer than the previous 9 weeks (up to 6 months) and therefore, these results should be compared with caution. In addition, with regards to the mutant mice, we found a strong survival bias, as the mice with preserved function tended to survive through more pregnancies. The current study design, and the use of mice at older time points, probably contributed to the observed survival bias. As it pertains to cardiac structure, pregnancy has been well documented in humans to induce physiological hypertrophy and increase left ventricular volume, though the effect of pregnancy in mice is less clear [42–45]. In our study, we found that both the WT and the Hsp20-S10F groups develop dilation and subsequently higher stroke volume and cardiac output after two pregnancies, however in the absence of a non-pregnant control it is possible that some of the observed effects may be age related and only the untreated S10F group demonstrated an increase in LV mass. These findings are consistent with not only cavity dilation, but also with LV remodeling that is commonly observed during the development of heart failure [46].

The Hsp20-S10F PPCM model also had significantly increased mRNA levels of ANP, consistent with the development of pathological hypertrophy. Additionally, TRPV2 has been found by our laboratory, as well as others, to be a stretch activated channel that increases expression under stress/stretch conditions [47,48]. In this model, we found that TRPV2 expression did not change in WT mice, but was significantly higher in untreated Hsp20-S10F mice in comparison to the treated which is consistent with the observed LV remodeling in the untreated mice.

Furthermore, it has been previously demonstrated that Hsp20 promotes angiogenesis via VEGF (though the effect of the S10F mutation in VEGF expression has yet to be published)[49]. We found that VEGF was increased in the treated mice, a finding that is congruent with the current fund of knowledge that impaired VEGF signaling is associated with cardiac dysfunction in mice. However, late pregnancy is a strong anti-angiogenic environment, in part due to the secretion by the placenta of anti-angiogenic factors like a sFlt1 that bind to and neutralize soluble members of the VEGF family [8]. In contrast, we have found that probenecid increases VEGF post-pregnancy and while the mechanism in unclear, the outcome in our model was significantly improved.

Thus, while the precise mechanism of action of probenecid in this mouse model was not the subject of investigation, it may be the case that TRPV2 activation reduced pathologic remodeling, possibly through improved calcium cycling in this model with impaired calcium handling [21] or potentially through increased VEGF production. These findings are still of course preliminary. Further, the generalizability of the TRPV2 findings to human population will require further study since even though our data in animals demonstrated increased TRPV2 expression in response to stress [47], some researchers have reported decreased TRPV2 expression in human subjects with heart failure[50], while others have found no difference [51].

While Hsp20, under physiologic conditions, protects against apoptosis [20], mutations, such as P20L [20] and S10F [14], abrogate these cardioprotective effects. The untreated Hsp20-S10F mice in our study had similar decreases in the Bcl-2/Bax and p-Akt/t-Akt ratios. Interestingly, we found that probenecid therapy had an antiapoptotic effect via increasing the Bcl-2/Bax pathway ratio. Furthermore, probenecid treatment decreased Akt phosphorylation, with no change in total Akt expression in both the WT and the Hsp20-S10F mice, decreasing the overall ratio of p-Akt/t-Akt. However, since the p-Akt/t-Akt ratio was also decreased in the Hsp20-S10F control mice, there are likely multiple other factors involved in the regulation of apoptosis in this and other models of cardiomyopathy. Previous studies suggested blockade of PANX1 channels by probenecid may increase the incidence of apoptosis in vitro and ex vivo [24,52]. In fact, Vessey et al. found that blockade of PANX1 channels by carbenoxolone leads to a decrease in phosphorylated Akt and a subsequent increase in apoptosis in an ex vivo model of ischemic preconditioning, but did not report on changes in Bcl-2/Bax [52]. Similarly, Poon et al. found that probenecid increases the number of apoptotic bodies in Jurkat cells and increases the number of apoptotic bodies with reduced cellular complexity in thymocytes [24]. In our current study, it may be inferred that probenecid induced apoptosis in the Hsp20-S10F mutant treated group by blocking PANX1 channels, though the levels of phosphorylated Akt were not significantly affected, while Bcl-2/Bax was preserved to control levels.

Lastly, while Fan et al. found that overexpression of Hsp20 reduced interstitial fibrosis [16], we found that there was no change to the level of fibrosis in any of the hearts, suggesting that probenecid treatment did not appear to have any positive or negative effects. Furthermore, the levels of fibrosis were not previously reported in the Hsp20 mutations of P20L or S10F [14,20] making comparisons between our studies and the previously published studies difficult.

### Conclusions and future directions

In summary, our data demonstrate that probenecid improves survival in a novel mouse model of PPCM by improving contractility and reducing LV remodeling, possibly through regulation of calcium cycling and less likely through apoptotic signaling pathways. The blunting of remodeling by probenecid during physiologic pregnancies without adverse effects on survival will require further study as will the potential clinical implications of our findings. Future clinical studies in patients with PPCM may warrant analysis for mutations in the Hsp20, which may potentially inform treatment options for those patients (i.e probenecid, bromocriptine, or maybe both).

## Supporting information

S1 Checklist NC3Rs ARRIVE guidelines checklist Onusko. Koch, Sheryl. 2020. “Probenecid Treatment Improves Outcomes in a Novel Mouse Model of Peripartum Cardiomyopathy.” OSF. March 5. osf.io/37csg.
(PDF)

## Abbreviations

DCM: Dilated cardiomyopathy
EF: Ejection fraction
GAPDH: Glyceraldehyde 3-phosphate dehydrogenase
HFrEF: Heart failure with reduced ejection fraction
Hsp20: Heat shock protein 20
PPCM: Peripartum cardiomyopathy
qRT-PCR: Quantitative Reverse transcription polymerase chain reaction
TRPV2: Transient receptor potential vanilloid 2
